# Clinical correlation of hemodynamic parameters using blood flow prediction model in cerebrovascular arteries

**DOI:** 10.3389/fphys.2026.1874450

**Published:** 2026-10-02

**Authors:** Jun Yup Kim, Wheesung Lee, Young-Lin Yoo, Da Hyun Lee, Si Un Lee, Beom Joon Kim, Jihoon Kang, Chae-Yong Kim, Tae-Rin Lee, Kihwan Hwang

**Affiliations:** 1 Department of Neurology, Seoul National University Bundang Hospital, Seoul National University College of Medicine, Seongnam, Republic of Korea; 2 NEAR Brain Inc., Seoul, Republic of Korea; 3 Department of Radiology, Seoul National University Bundang Hospital, Seoul National University College of Medicine, Seongnam, Republic of Korea; 4 Department of Neurosurgery, Seoul National University Bundang Hospital, Seoul National University College of Medicine, Seongnam, Republic of Korea

**Keywords:** cerebrovascular hemodynamics, computational blood flow modeling, non-invasive optimal vessel analysis, patient-specific vascular modeling, time-of-flight magnetic resonance angiography

## Abstract

**Introduction:**

Patient-specific hemodynamic assessment is pivotal for optimizing treatment strategies in cerebrovascular interventions; however, conventional modalities are often limited by restricted clinical accessibility or prolonged acquisition times.

**Methods:**

This study evaluates the previously developed Dr. NEAR flow (DNF), a streamlined computational framework designed for rapid and clinically feasible blood flow prediction, validated against Non-invasive Optimal Vessel Analysis (NOVA), a quantitative magnetic resonance angiography technique. The DNF methodology reconstructs a one-dimensional vascular topology from standard time-of-flight magnetic resonance angiography (MRA-TOF) sequences, and flow rate, velocity, and relative pressure are computed throughout the reconstructed vascular network using a Poiseuille-based fluid model. For validation, patient-specific boundary conditions were parameterized using proximal inflow measurements obtained from NOVA, and quantitative comparisons were performed at arterial locations with corresponding NOVA measurements.

**Results:**

Across 230 arterial segments in 35 subjects, NOVA-measured and DNF-predicted flow rates were strongly correlated (*r* = 0.77), and the association remained significant after accounting for within-patient clustering (*β* = 0.863, 95% CI 0.769–0.957). Repeated-measures Bland–Altman analysis showed a mean bias (−58.04 mm³/s). After conversion of flow rates to velocities, vessel-averaged distributions across the major cerebral arteries showed generally similar patterns between the two modalities, although artery-specific correlations were variable.

**Discussion:**

These findings indicate that DNF is a feasible approach for cerebrovascular hemodynamic assessment.

## 1 Introduction

Patient-specific blood flow within the cerebrovascular network provides essential information for optimizing treatment planning in surgical and endovascular interventions (Liebeskind et al., 2015; Kern and Liebeskind, 2019). Despite this clinical need, direct measurement of cerebral blood flow remains challenging due to limited accessibility. In clinical practice, transcranial Doppler ultrasound (TCD) is widely used for real-time assessment of blood flow patterns in cerebral arteries (Aaslid et al., 1982). However, TCD is highly operator-dependent and is limited in its ability to evaluate small or deeply located vessels (Purkayastha and Sorond, 2012). To overcome these limitations, various magnetic resonance–based techniques have been developed: Non-invasive Optimal Vessel Analysis (NOVA), which uses targeted 2D phase-contrast (PC) MRI for vessel-specific quantitative flow measurement across prescribed vessel cross-sections (Khan et al., 2017), time-averaged 3D PC MRI, which enables volumetric measurement of three-directional blood-flow (MacDonald et al., 2016), and 4D flow MRI, which provides time-resolved three-dimensional velocity information over a vascular volume for visualization of complex hemodynamic patterns (Aristova et al., 2019). The prolonged acquisition time and extensive post-processing required for 4D flow MRI, however, limit routine clinical application (Morgan et al., 2021).

As an alternative, computational fluid dynamics (CFD) has been extensively investigated for predicting hemodynamics in patient-specific cerebrovascular models (Grinberg et al., 2009; Cho et al., 2022). These approaches provide detailed spatial distributions of velocity and pressure that are difficult to obtain in clinical settings. However, a major limitation of CFD is its strong dependence on assumed boundary conditions, which are often uncertain in real-world clinical practice. In addition, CFD simulations are computationally intensive and require substantial processing time. To address these limitations, Pegolotti et al. (2024) recently proposed a graph neural network (GNN)–based model. Using skeletonized graph representations of the cerebrovascular system, GNN algorithms were employed to predict blood flow patterns. Although such computational approaches demonstrate potential clinical applicability, they remain largely confined to the research domain due to challenges in integration with existing clinical workflows.

Dr. NEAR flow (DNF), a simplified and clinically feasible flow prediction framework, was previously developed and evaluated in our previous study (Lee et al., 2026). In that study, DNF-derived cerebral blood flow velocities were compared with TCD measurements using patient-specific TCD-derived inflow conditions. TCD, however, primarily provides localized velocity measurements, requiring volumetric flow rate to be estimated using MRA-derived vessel cross-sectional areas. In contrast, NOVA provides vessel-specific volumetric flow measurements directly from phase-contrast MRI. The present study therefore extends the previous evaluation by assessing DNF against NOVA-derived quantitative volumetric flow measurements in a separate clinical cohort. DNF represents the cerebrovascular system as a graph extracted from standard time-of-flight magnetic resonance angiography (MRA-TOF) data and predicts blood flow rate, velocity, and relative pressure using a linear Poiseuille flow model with patient-specific proximal inflow measurements obtained from NOVA as boundary conditions. Unlike 3D MRI or 4D flow MRI, which directly acquires volumetric velocity information, DNF computationally extends this proximal flow information throughout the reconstructed vascular network, including arterial segments without direct PC MRI measurements. The predicted flow rates are compared with those obtained using NOVA in the major cerebral arteries. Pearson correlation analysis and Bland–Altman (Bland and Altman, 2007) plots are employed to quantify the agreement between the proposed method and conventional flow measurements. Furthermore, the blood flow pattern change through stenosis region is extracted from DNF results to show its applicability on stenosis cases. Additionally, the differences of blood flow pattern by subgroups, *i.e.* diabetes, dyslipidemia, and hypertension, are investigated by NOVA and DNF results.

## 2 Methods

### 2.1 Patient population

This retrospective observational study included patients evaluated in the outpatient neurology clinic for cerebrovascular disease at Seoul National University Bundang Hospital between March 2021 and June 2024. Eligible patients were those who underwent NOVA and MRA-TOF during the study period. To ensure temporal consistency of hemodynamic assessment, NOVA and MRA-TOF examinations were required to be performed within 6 months of each other. Patients were included if NOVA flow measurements and corresponding MRA-TOF datasets were available and of sufficient quality for computational vascular reconstruction and analysis. Patients with incomplete imaging data or inadequate image quality precluding reliable vascular segmentation were excluded. A total of 35 patients met the eligibility criteria and were included in the final analysis. The study protocol was approved by the Institutional Review Board of Seoul National University Bundang Hospital (IRB No. B-2512-1013-104). The requirement for informed consent was waived by the Institutional Review Board due to the retrospective nature of the study.

### 2.2 Demographics and clinical variables

Demographic and clinical data were obtained from electronic medical records. Collected variables included age, sex, vascular risk factors (hypertension, diabetes mellitus, dyslipidemia, and smoking status), history of stroke, and coronary heart disease. Physiological parameters, including height, weight, body mass index, and resting blood pressure, were recorded at the time of imaging. Information regarding ongoing medical therapy, including statins, antiplatelet agents, and anticoagulants, was also documented.

### 2.3 Imaging acquisition of MRA-TOF

All imaging was performed on a 3T MRI scanner (Ingenia CX; Philips Healthcare, Best, The Netherlands) using a 32-channel head coil. 3D TOF MRA was performed using a fast field echo (FFE) sequence. The imaging parameters were as follows: repetition time (TR)/echo time (TE) = 23/3.45 ms; flip angle = 18°; field of view (FOV) = 180 × 180 × 84 mm³; acquisition voxel size = 0.6 × 0.74 × 0.8 mm³; and reconstruction voxel size = 0.34 × 0.34 × 0.4 mm³. A total of 210 transverse slices were acquired in five overlapping chunks using a parallel imaging (SENSE) factor of 2.5 in the right-left direction. To optimize signal intensity, a tilted optimized non-saturating excitation (TONE) pulse was applied with a ramp angle starting at 16.2°. A superior saturation band (20 mm thickness, 10 mm gap) was used to suppress venous flow, and flow compensation was enabled. The total acquisition time was 5 minutes 47 seconds.

### 2.4 Flow measurement using NOVA

Quantitative blood flow analysis was performed using NOVA software. NOVA provides vessel-specific volumetric flow measurements using targeted two-dimensional PC MRI. Target vessel measurement planes were prescribed perpendicular to the vessel axis, guided by 3D TOF-MRA. PC imaging employed a T1-weighted FFE sequence with turbo field echo (TFE) readouts. Key parameters included a 15°flip angle, 4–5 mm slice thickness, and an acquisition matrix ranging from 192 × 96 to 196 × 142. Velocity encoding (VENC) and timing parameters were tailored to each vascular segment to optimize signal-to-noise ratio and prevent aliasing. VENC was set to 100 cm/s (TR/TE = 6.9–7.4/4.4–4.8 ms) for the internal and common carotid arteries, and 100 cm/s (TR/TE = 8.1/5.3 ms) for the basilar artery. For the middle, posterior, and anterior cerebral arteries, VENC ranged between 80 and 100 cm/s (TR/TE = 7.7–9.0/4.9–6.0 ms). Cardiac synchronization was achieved via peripheral pulse unit with retrospective gating, capturing 10–13 phases of the pulsatile cycle. Flow compensation was applied to minimize motion artifacts. The software semi-automatically segmented vessel boundaries and derived volumetric flow rates (mL/min) by integrating velocity profiles over the cross-sectional areas.

### 2.5 Reconstruction of cerebral arteries using DNF

Computational blood flow analysis was performed using Dr. NEAR flow (NEAR Brain Inc., Korea), a computational software framework based on the one-dimensional cerebrovascular network model described in our previous study (Lee et al., 2026). The analysis workflow, as summarized in **Figure 1**, comprised three major stages: vessel segmentation and refinement, skeletonization, and blood flow calculation. MRA-TOF images, provided in DICOM format, were imported into the software and preprocessed using Gaussian smoothing with a standard deviation of 0.4 voxels to reduce noise. Voxel extraction was performed by thresholding the normalized volume at 2.55 standard deviations above the background mean. Coordinates of voxels exceeding this threshold were converted into a 3D point cloud, which was subsequently refined using Density-Based Spatial Clustering of Applications with Noise (DBSCAN) with a maximum neighborhood distance of 1.8 voxels and a minimum neighborhood size of 2 points to eliminate spatial outliers and non-vascular regions (van der Walt et al., 2014). To construct a graph representation of the vascular tree, each retained voxel was analyzed for topological connectivity with its neighbors. Voxels were classified as terminal, branch, or intermediate nodes depending on their connectivity degree (Hagberg et al., 2007). A graph structure was then generated by linking neighboring voxels that shared the same cluster label, forming a network of nodes and edges. Vessel radius at each centerline node was estimated from the reconstructed vascular surface extracted using marching cubes, with the radius defined as the mean Euclidean distance from the node to the five nearest surface vertices in physical coordinates. The final graph stored node identifiers, spatial coordinates, and effective vessel radii, alongside connectivity information describing the vascular topology.

**Figure 1:**
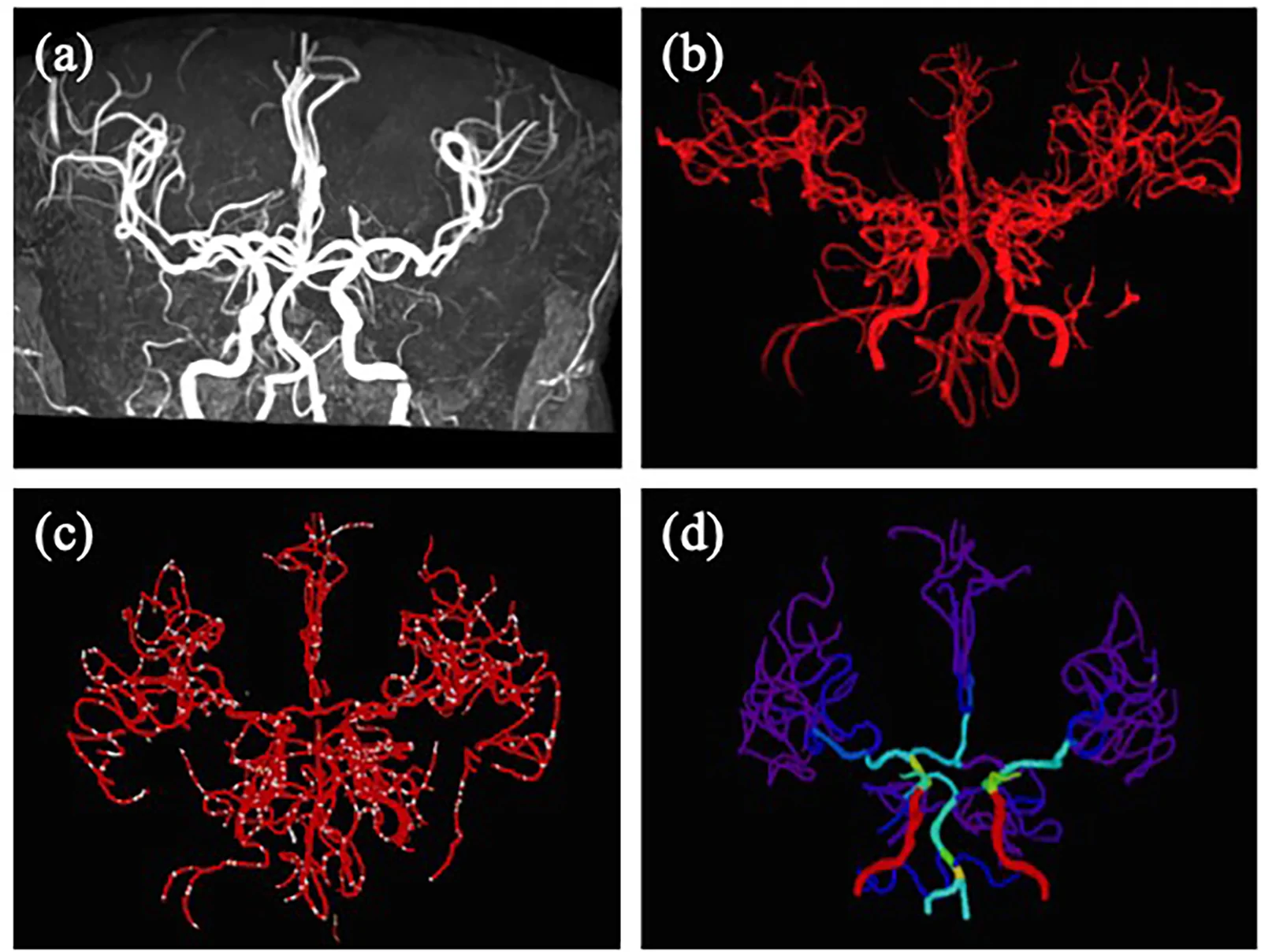
Workflow of hemodynamics prediction process. **(a)** Maximum-intensity-projection (MIP) image of the MRA-TOF dataset, **(b)** three-dimensional vessel segmentation, **(c)** 1D network reconstruction, and **(d)** computational flow rate simulation results.

### 2.6 Blood flow prediction using DNF

The blood flow rate through each vessel segment was modeled based on the Poiseuille flow assumption, represented by the following equation:


$$Q=\frac{\pi {\bar{r}}^{4}}{8\eta L}\;\text{Δ}P$$

where Q is the volumetric flow rate, $\bar{r}$ is the vessel radius, η is the blood viscosity, L is the vessel length, and ΔP is the pressure drop between the *i*-th and *j*-th nodes. Blood viscosity was assumed to be constant at $4.265\times 10^{-3}$ Pa·s for all patients, consistent with the previous DNF implementation (Lee et al., 2026), and was not adjusted according to hematocrit or other patient-specific physiological parameters. A fixed pressure of 10,000 Pa was prescribed at the distal outlet nodes as the reference pressure, and the resulting pressure values were reported as relative pressure within the vascular network. The vascular geometry used in the flow calculation was reconstructed from MRA-TOF, while patient-specific inflow boundary conditions were prescribed using flow rates measured by NOVA at the internal carotid and vertebral arteries. NOVA measurements prescribed as inflow boundary conditions were not considered independent validation targets. In patients for whom the basilar artery was used as the posterior-circulation inflow, the corresponding basilar artery measurement was excluded from the downstream comparison. Based on the computed flow rates and radii, the flow velocity at each node was calculated by dividing the flow rate by the cross-sectional area (*A*), where $A=\pi {\bar{r}}^{2}$. The final output of the analysis included the blood flow rate, velocity, and relative pressure distributions across the entire arterial network.

### 2.7 Statistical analysis

Statistical analyses were conducted to compare the blood flow rates and velocities of the major cerebral arteries within the Circle of Willis as calculated by DNF against those measured by NOVA, which served as the reference standard. The linear association between NOVA and DNF flow rates was summarized descriptively using Pearson’s correlation coefficient. Pearson correlations were also calculated separately for each artery, with each patient contributing at most one observation per artery. To account for multiple arterial measurements within each patient, the overall association between NOVA and DNF flow rates was evaluated using a linear mixed-effects model with DNF flow rate as the outcome, NOVA flow rate as a fixed effect, and patient as a random intercept. Artery type was additionally included as a categorical fixed effect in a sensitivity analysis. Agreement was assessed using a repeated-measures Bland–Altman variance-component model (Bland and Altman, 2007) with patient as a random intercept. The 95% limits of agreement were derived from the between-patient and within-patient variance components. For velocity comparison, NOVA-derived velocity was calculated by dividing the measured flow rate by the corresponding vessel cross-sectional area obtained from DNF vascular reconstruction. For DNF, a representative value for each arterial segment was derived by averaging the individual nodal velocities within that segment, resulting in 230 data points for each method. Furthermore, the distribution and physiological ranges of these vessel-averaged velocities across major arteries were visualized using box-and-whisker plots to compare the median values and variability between DNF and NOVA.

In addition, subgroup analyses were performed to investigate whether the agreement between NOVA and DNF differed according to major vascular risk factors, including diabetes mellitus, dyslipidemia, and hypertension. For each risk factor, patients were divided into two groups based on the presence or absence of the condition. Flow rates measured by NOVA and predicted by DNF were compared using a linear mixed-effects model including method, risk-factor status, and their interaction as fixed effects and patient as a random intercept. The method-by-risk-factor interaction assessed whether the DNF−NOVA difference varied according to risk-factor status. All linear mixed-effects models were fitted using restricted maximum likelihood estimation (Lindstrom and Bates, 1988). Fixed-effect regression coefficients (*β*) were reported with Wald 95% confidence intervals (CIs) and two-sided *p*-values, with *p* < 0.05 considered statistically significant. All statistical analyses were performed using Python version 3.12.13 with SciPy version 1.18.0 and statsmodels version 0.14.6.

## 3 Results

### 3.1 Patient characteristics

The baseline characteristics of the study population are summarized in **Table 1**. A total of 35 patients were included in the analysis. The mean age was 60.7 ± 17.3 years, and 7 patients (20.0%) were male. The mean body mass index (BMI) was 25.2 ± 3.0 kg/m². The mean systolic and diastolic blood pressures were 131.2 ± 13.8 mmHg and 73.5 ± 11.9 mmHg, respectively. Regarding vascular risk factors, hypertension was present in 27 patients (77.1%), dyslipidemia in 23 (65.7%), and diabetes mellitus in 19 (54.3%). A history of prior stroke was observed in 33 patients (94.3%), and coronary heart disease was documented in 11 patients (31.4%). Twenty patients (57.1%) were current smokers. With respect to medical therapy, 34 patients (97.1%) were receiving statins, all patients (100.0%) were on antiplatelet therapy, and 4 patients (11.4%) were treated with anticoagulants at the time of evaluation.

**Table 1:** Patient characteristics.

Variable	Value (n=35)
Age, mean ± SD (years)	60.7 ± 17.3
Male sex, n (%)	7 (20.0)
BMI, mean ± SD (kg/m²)	25.2 ± 3.0
Systolic BP, mean ± SD (mmHg)	131.2 ± 13.8
Diastolic BP, mean ± SD (mmHg)	73.5 ± 11.9
Past medical history	
Diabetes mellitus, n (%)	19 (54.3)
Dyslipidemia, n (%)	23 (65.7)
Hypertension, n (%)	27 (77.1)
Stroke, n (%)	33 (94.3)
Coronary heart disease, n (%)	11 (31.4)
Smoking, n (%)	20 (57.1)
History of medication	
Statin, n (%)	34 (97.1)
Antiplatelets, n (%)	35 (100)
Anticoagulants, n (%)	4 (11.4)

n, number of subjects and SD, standard deviation.

### 3.2 Computational flow analysis results

Computational modeling using DNF was successfully performed in 35 patients, yielding comprehensive blood flow rate distributions across the arterial network. This was achieved through DNF’s integrated computational workflow, encompassing 3D reconstruction and subsequent blood flow prediction, as illustrated in **Figure 1**. Patient-specific boundary conditions were established at the internal carotid and vertebral arteries (**Figure 2**). In 14 patients in whom bilateral vertebral artery flow signals were not detectable on MRA-TOF images, proximal inflow conditions were instead applied at the basilar artery inlet. Accordingly, the basilar artery comparison in **Table 2** included the remaining 21 patients, in whom the vertebral arteries were used as posterior-circulation inflow boundaries. Based on these individualized datasets, DNF generated comprehensive hemodynamic outputs, including pressure differences, flow rates, and velocities (**Figure 3**). A total of 230 arterial-level flow rate pairs were included in the final comparative analysis. One data point involving the right anterior cerebral artery was excluded due to the absence of a corresponding NOVA flow measurement.

**Figure 2:**
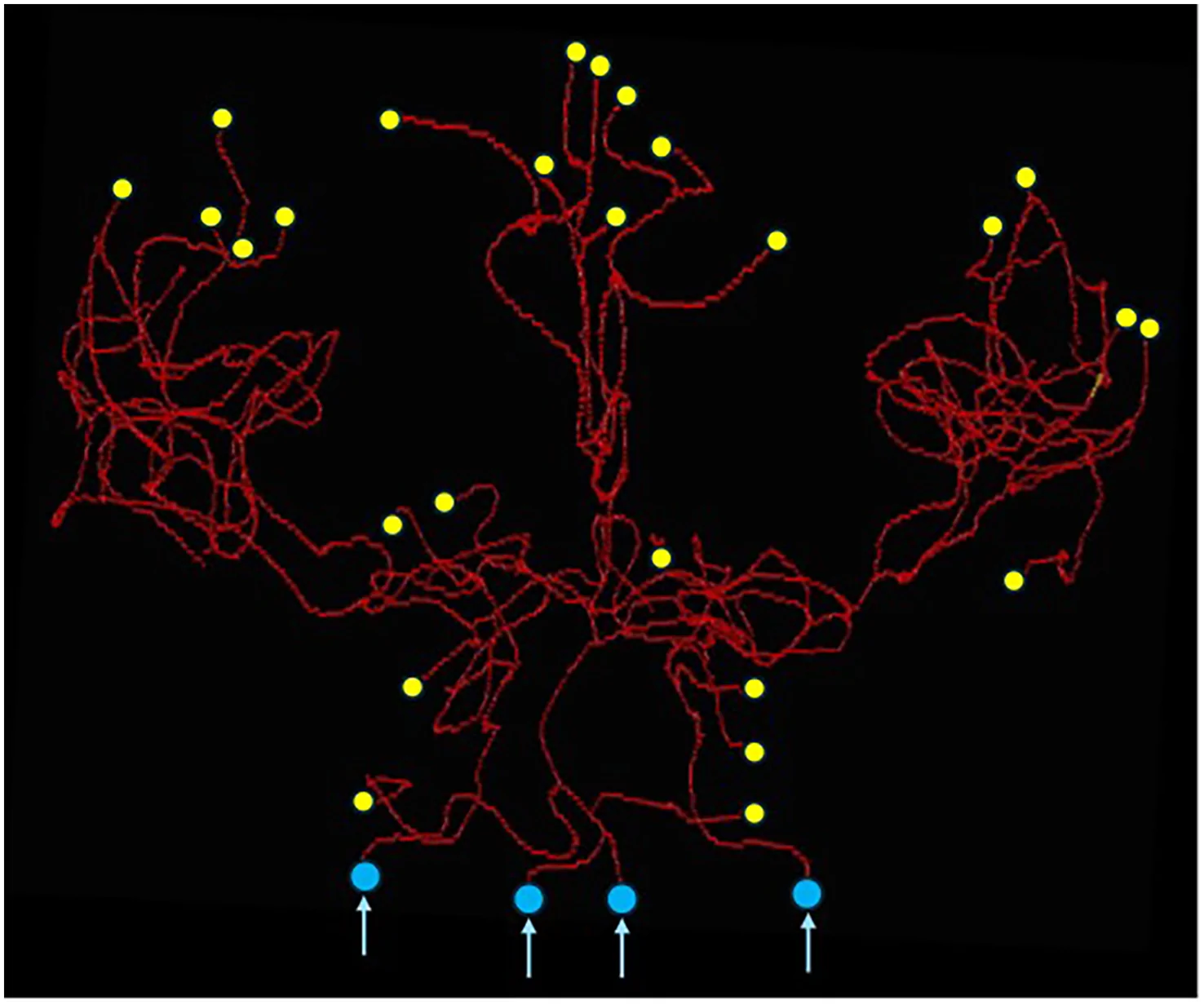
Computational graph model. Flow simulation is performed on a connected 1D network where boundary conditions are defined at inlets (blue dots) and outlets (yellow dots).

**Table 2:** Artery-specific comparison of flow rates between NOVA and DNF.

Artery	n	NOVA (mm³/s)	DNF (mm³/s)	*r*	*p*
Basilar Artery	21	3113.49 ± 1692.52	3750.86 ± 1696.20	0.95	< 0.0001
Left Posterior Cerebral Artery – P2	35	1241.90 ± 512.80	1372.52 ± 669.46	0.67	< 0.0001
Right Posterior Cerebral Artery – P2	35	1368.57 ± 658.28	1307.85 ± 734.19	0.64	< 0.0001
Left Anterior Cerebral Artery	35	1772.86 ± 842.44	1537.89 ± 867.96	0.35	0.0393
Right Anterior Cerebral Artery	34	1530.88 ± 825.35	1484.08 ± 877.47	0.73	< 0.0001
Left Middle Cerebral Artery	35	2678.10 ± 803.94	2232.93 ± 1102.64	0.68	< 0.0001
Right Middle Cerebral Artery	35	2447.14 ± 806.65	2339.05 ± 1005.10	0.48	0.0035

Values are presented as mean ± standard deviation. NOVA, Non-invasive Optimal Vessel Analysis; DNF, Dr. NEAR flow; n, number of arteries; *r*, Pearson’s correlation coefficient; *p*, statistical significance.

**Figure 3:**
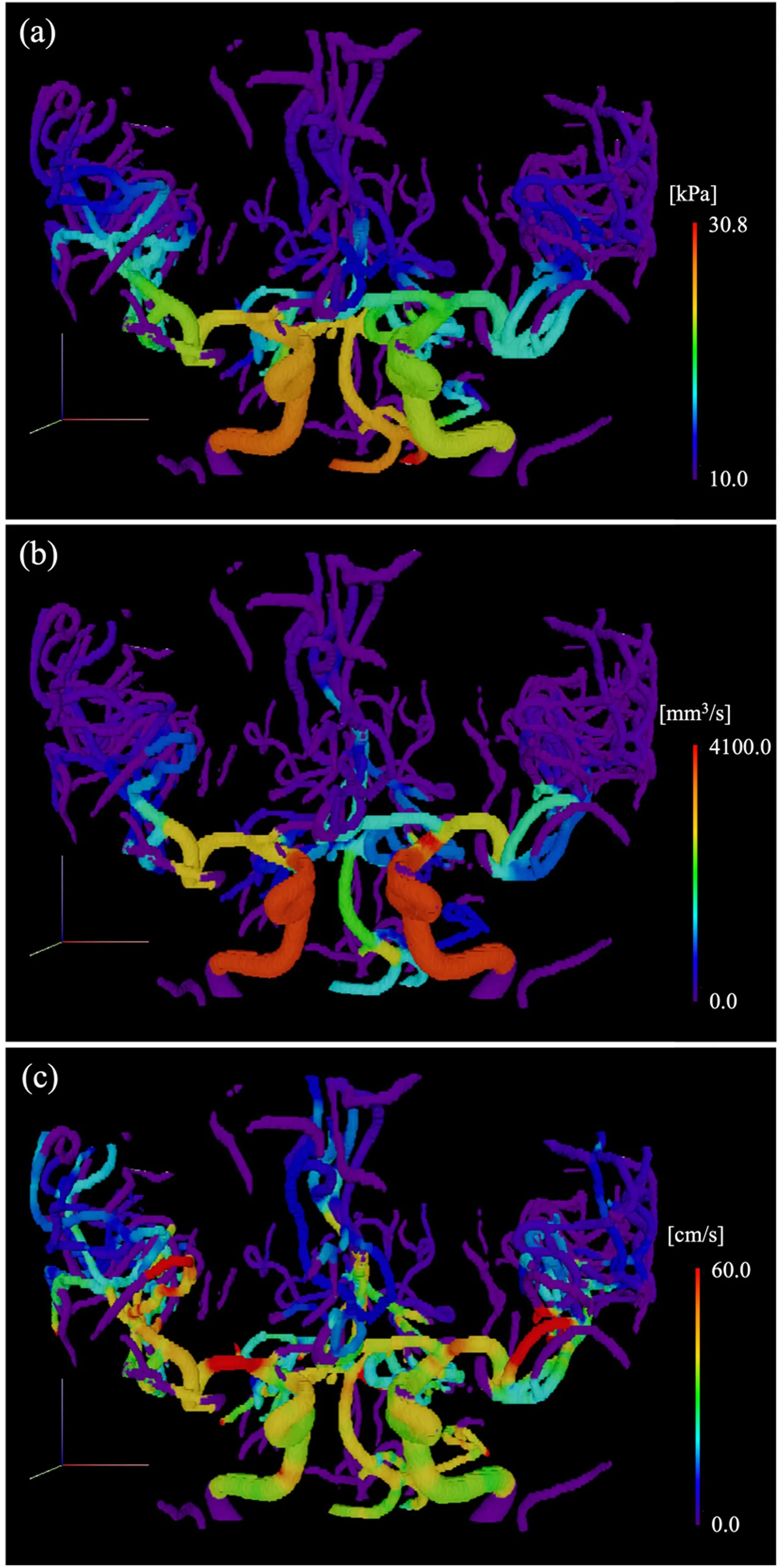
Predicted hemodynamic parameters in a cerebrovascular network. Dr. NEAR flow calculates **(a)** relative pressure, **(b)** flow rate, and **(c)** velocity based on vascular geometry reconstructed from MRA -TOF and patient-specific proximal inflow boundary conditions obtained from NOVA.

### 3.3 Comparative analysis of hemodynamic distribution and quantitative agreement

To evaluate the clinical utility and spatial coverage of the proposed model, the hemodynamic distributions predicted by DNF were visualized alongside those obtained from NOVA. **Figure 4** illustrates the flow rate and velocity distributions across the cerebral arterial network. In this comparison, NOVA-derived velocities were calculated by dividing measured flow rates by vessel cross-sectional areas extracted from the DNF model. Whereas NOVA provides flow measurements primarily in the major arteries of the Circle of Willis, DNF enabled calculation of hemodynamic variables across a more extensive vascular tree. As demonstrated in the contour plots, DNF extended flow analysis to distal arterial segments where NOVA measurements were unavailable, thereby providing continuous velocity and relative pressure distributions throughout the reconstructed network.

**Figure 4:**
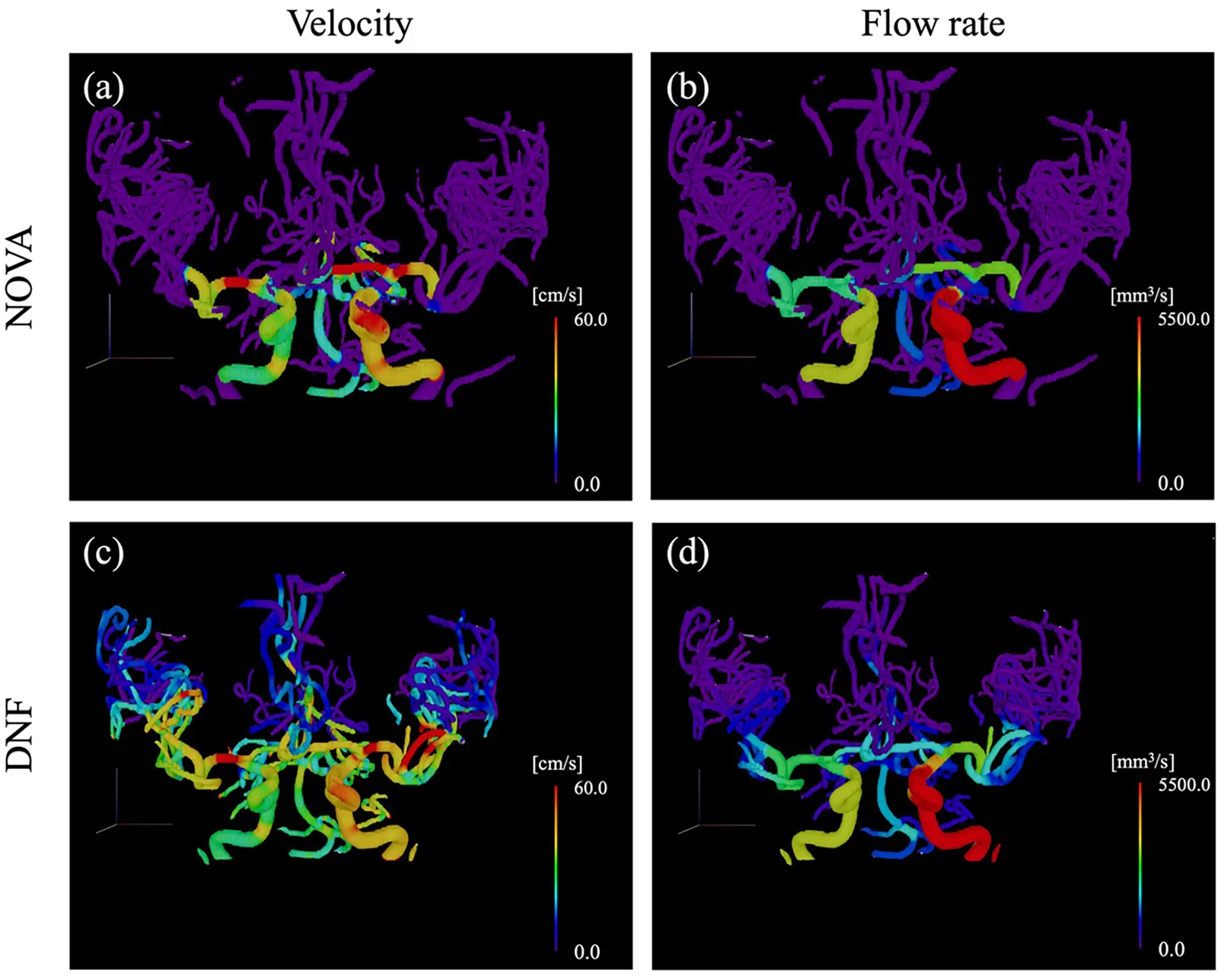
Qualitative comparison between non-invasive optimal vessel analysis (NOVA) and Dr. NEAR flow (DNF). Contour plots represent **(a)** NOVA-derived velocity and **(b)** measured flow rates, alongside **(c)** DNF-predicted velocity and **(d)** predicted flow rates.

To evaluate the agreement of these calculations with *in vivo* flow measurements, DNF-predicted flow rates were compared with NOVA measurements across the seven major cerebral arteries, including the basilar artery and the bilateral posterior cerebral, anterior cerebral, and middle cerebral arteries. A strong positive correlation was observed between the two methods. Pearson correlation analysis demonstrated a strong linear association (descriptive *r* = 0.77; **Figure 5**), which remained significant after accounting for within-patient clustering using a patient-level random-intercept linear mixed-effects model (*β* = 0.863, 95% CI 0.769–0.957, *p* < 0.0001). The association also remained significant after adjustment for artery type (*β* = 0.774, 95% CI 0.664–0.883, *p* < 0.0001). Repeated-measures Bland–Altman analysis showed a mean bias of −58.04 mm³/s and 95% limits of agreement [−1596.77, 1480.70] mm³/s. A total of 216 of 230 observations (93.91%) fell within these limits (**Figure 6**). A significant proportional bias was also observed (slope = 0.131, 95% CI 0.037–0.225, *p* = 0.006).

**Figure 5:**
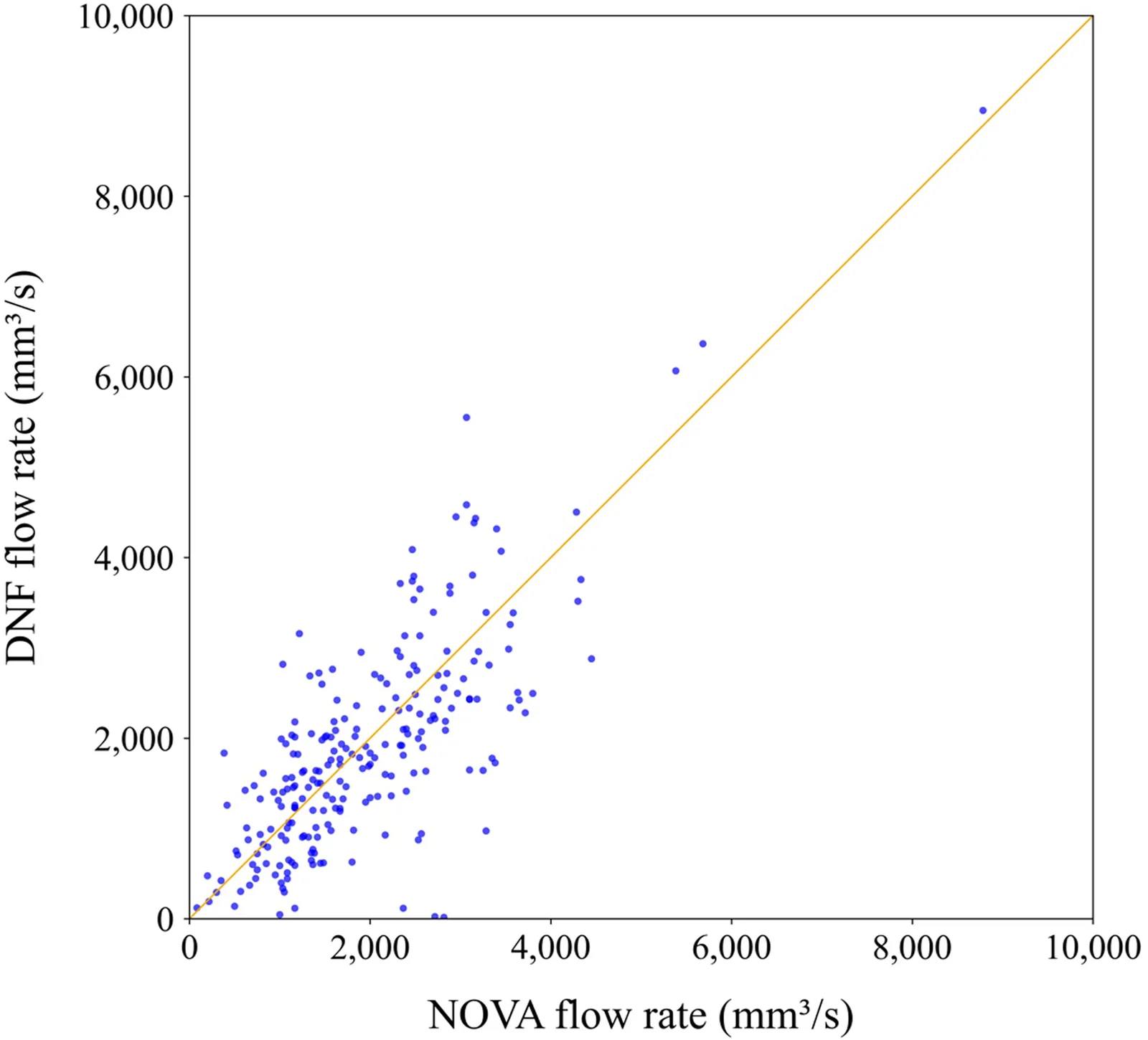
Pearson correlation analysis of flow rates between non-invasive optimal vessel analysis (NOVA) and Dr. NEAR flow (DNF).

**Figure 6:**
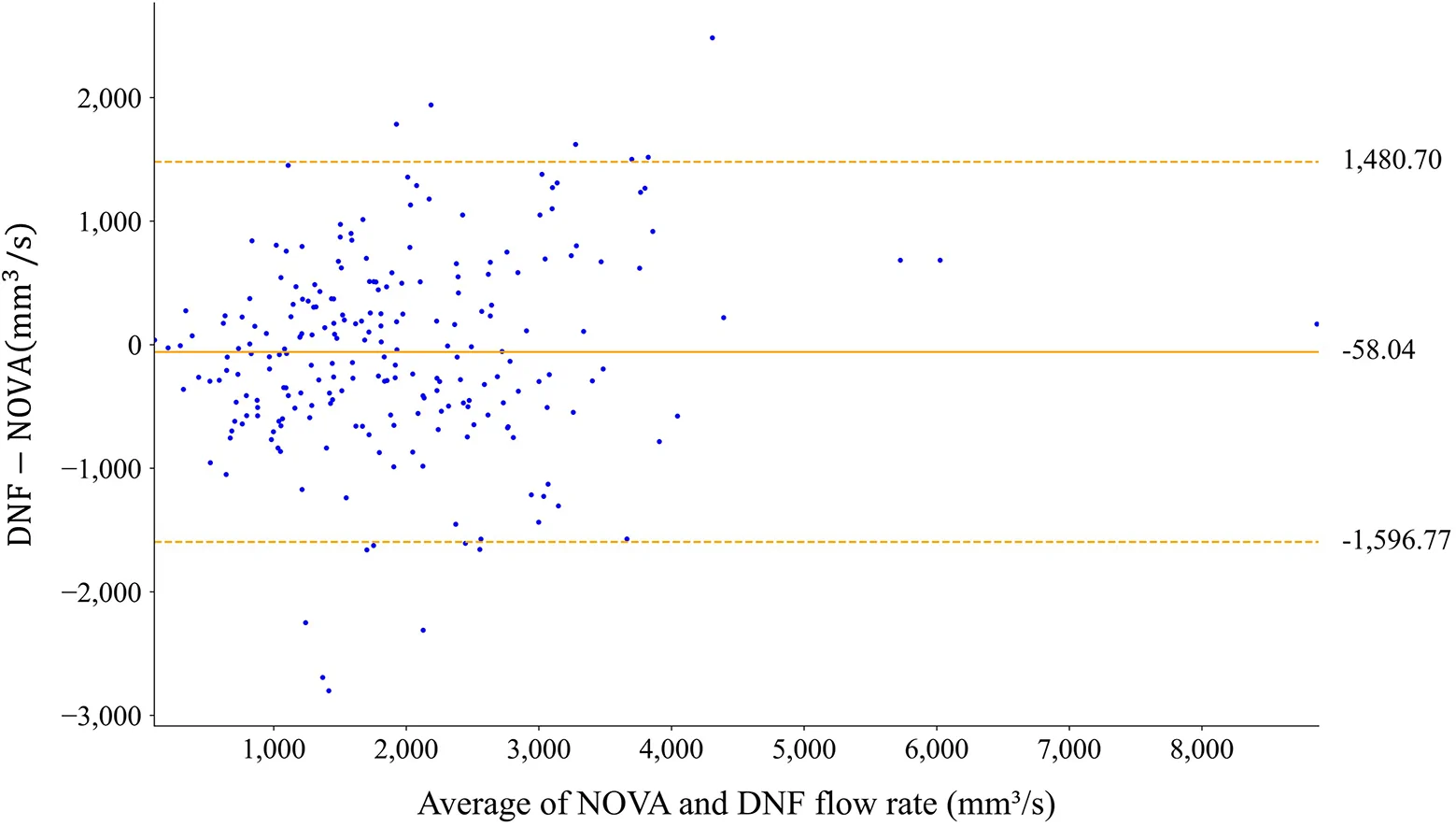
Repeated-measures Bland–Altman plot for flow rate agreement between non-invasive optimal vessel analysis (NOVA) and Dr. NEAR flow (DNF).

### 3.4 Artery-specific comparisons

Artery-specific comparisons of NOVA-measured and DNF-predicted flow rates are summarized in **Table 2**. Moderate-to-strong correlations in flow rates between NOVA and DNF were observed across individual arteries, with correlation coefficients ranging from *r* = 0.35 to *r* = 0.95. The strongest correlation was identified in the basilar artery, whereas the left anterior cerebral artery demonstrated the lowest correlation. Each patient contributed at most one observation to each artery-specific analysis. Consequently, the correlations did not include repeated observations from the same patient within a given artery.

## 4 Discussion

A primary advantage of this approach is its computational efficiency. Although high-fidelity three-dimensional CFD models offer detailed spatial resolution, they require extensive pre-processing and substantial computational resources, which often limit their feasibility in time-sensitive clinical environments (Steinman, 2002; Yang et al., 2023). In contrast, the linearized one-dimensional network model provides a rapid alternative for cerebrovascular hemodynamic assessment. At the same time, the present study demonstrates that DNF, a computational modeling approach based on a linearized fluid dynamics framework, provides blood flow rate estimates that strongly correlate with NOVA measurements (*r* = 0.77), with the association remaining significant after accounting for repeated arterial measurements within patients. This overall correlation was similar in magnitude to the correlation of *r* = 0.74 previously observed between DNF-derived and TCD-measured velocities (Lee et al., 2026), although the two studies evaluated different hemodynamic quantities and reference modalities. Furthermore, 93.91% of observations fell within the limits of agreement in the repeated-measures Bland–Altman analysis. However, the wide limits of agreement and significant proportional bias indicate that caution is warranted when interpreting individual arterial estimates, particularly across different flow magnitudes.

For the purpose of validation, NOVA-derived measurements were intentionally used as input variables to assess the distributive accuracy and internal consistency of the DNF algorithm. By aligning proximal inflow conditions with an established clinical modality, this study isolated and verified the model’s ability to simulate downstream flow distribution throughout the arterial network. Compared with the previous TCD-based evaluation, which required conversion of measured velocity using MRA-derived vessel cross-sectional areas, the NOVA-based comparison provides an assessment at the volumetric flow-rate level, which is directly related to flow redistribution within the vascular network. Although the use of NOVA-derived measurements as proximal inflow conditions introduces methodological dependence on selected input vessels, these patient-specific boundary conditions enabled evaluation of downstream flow partitioning throughout the reconstructed vascular network. In addition to this methodological dependence, the absence of controlled phantom validation, as performed for related reduced-order Circle of Willis models (Cieslicki and Ciesla, 2005), remains a limitation of the present study. In future applications, controlled phantom experiments could provide independent validation of DNF under prescribed flow conditions, while full methodological independence could be achieved by integrating cardiac output and electrocardiographic data into mathematical models (Alastruey et al., 2008; Reymond et al., 2009) to estimate patient-specific inflow conditions. Such advancement would enable the computational workflow to function as a stand-alone tool, providing streamlined, patient-specific hemodynamic profiles derived solely from routinely available clinical parameters, even in settings where specialized flow-sensitive imaging is not accessible.

Despite the strong overall correlation, lower performance was observed in smaller vessels, such as the left anterior cerebral artery (*r* = 0.35). This discrepancy is primarily attributable to the spatial resolution limitations of MRA-TOF imaging; small vessels are represented by fewer voxels, increasing uncertainty in radius estimation. Given that flow in the Poiseuille equation is proportional to the fourth power of the radius, even minor segmentation inaccuracies are amplified in the resulting flow calculations (Hoogeveen et al., 1998). This inherent mathematical sensitivity likely explains the greater variance observed in smaller arterial segments compared with larger vessels such as the basilar artery (*r* = 0.95). In addition to this geometric sensitivity, the steady-state Poiseuille formulation does not account for pulsatility or secondary flow effects associated with curved and complex cerebrovascular geometries (Oshima et al., 2005; Chassagne et al., 2021). Therefore, DNF-derived velocity and relative pressure should be interpreted with these geometric and modeling limitations in mind.

Artery-specific comparisons of velocity distributions, illustrated in the box-and-whisker plots (**Figure 7**), showed variable agreement between DNF-predicted velocities and NOVA-derived values across major arterial segments. Quantitative comparisons of blood flow velocities between the two methods are summarized in **Table 3**. For vessel-level comparison, velocity values were calculated by averaging nodal velocities within each arterial segment for each subject, yielding 230 representative data points. The correlations between DNF-predicted and NOVA-derived velocities varied across arterial segments, with particularly weak and non-significant associations in the left anterior cerebral artery (*r* = 0.08, *p* = 0.6478) and right middle cerebral artery (*r* = 0.24, *p* = 0.1649). These artery-specific differences in velocity reflect not only the corresponding differences in flow rate but also the dependence of velocity on vessel cross-sectional area. Because the vessel geometry used for velocity calculation was reconstructed from MRA-TOF, uncertainties associated with image resolution, segmentation, and radius estimation can propagate into the calculated velocity. NOVA-derived velocities were also calculated using cross-sectional areas obtained from the DNF reconstruction, introducing methodological dependence into the velocity comparison.

**Figure 7:**
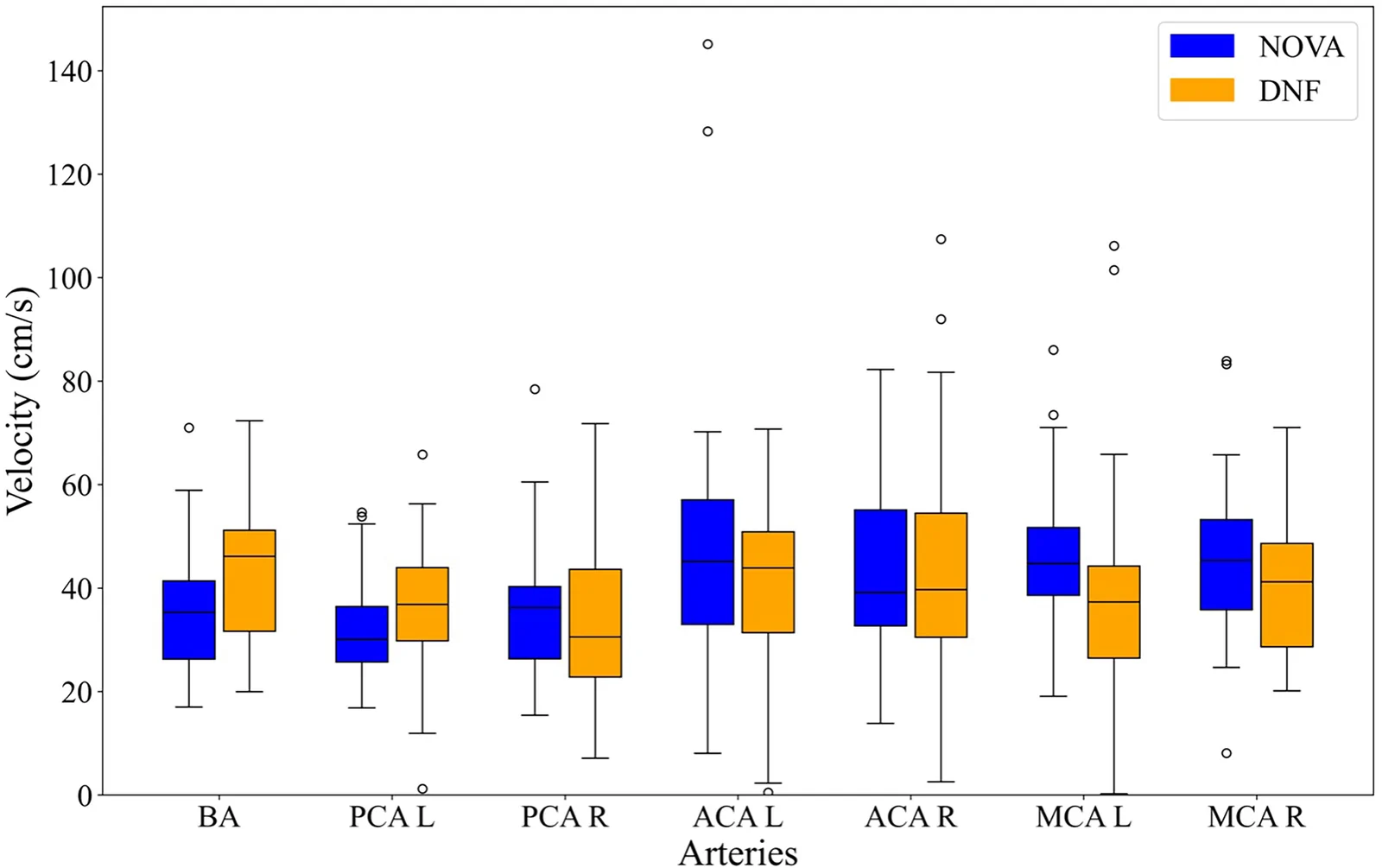
Comparison of vessel-averaged velocities between non-invasive optimal vessel analysis (NOVA) and Dr. NEAR flow (DNF) across major cerebral arteries.

**Table 3:** Comparison of blood flow velocities between NOVA and DNF.

Artery	n	NOVA (cm/s)	DNF (cm/s)	*r*	*p*
Basilar Artery	21	34.43 ± 13.15	41.90 ± 13.51	0.90	< 0.0001
Left Posterior Cerebral Artery – P2	35	30.66 ± 9.31	33.67 ± 13.72	0.43	0.0099
Right Posterior Cerebral Artery – P2	35	34.38 ± 12.33	32.37 ± 15.03	0.41	0.0144
Left Anterior Cerebral Artery	35	43.19 ± 20.24	37.00 ± 16.46	0.08	0.6478
Right Anterior Cerebral Artery	34	40.79 ± 16.41	39.90 ± 21.56	0.66	< 0.0001
Left Middle Cerebral Artery	35	44.11 ± 13.56	36.91 ± 20.26	0.74	< 0.0001
Right Middle Cerebral Artery	35	41.99 ± 13.43	38.84 ± 13.22	0.24	0.1649

Values are presented as mean ± standard deviation. NOVA, Non-invasive Optimal Vessel Analysis; DNF, Dr. NEAR flow; n, number of arteries; *r*, Pearson’s correlation coefficient; *p*, statistical significance.

An additional strength of DNF lies in its ability to provide comprehensive quantitative information in the presence of arterial stenosis or occlusion. Whereas conventional MRA primarily offers anatomical visualization of luminal narrowing and NOVA quantifies flow within selected arterial segments, the proposed model additionally computes pressure gradients and velocity alterations throughout the vascular tree (**Figure 8**). This is enabled by the network-based architecture, in which geometric attributes—such as vessel radius and spatial coordinates—are stored at each node. Based on these nodal parameters, hemodynamic resistance and local flow alterations resulting from vessel narrowing are propagated across interconnected edges. Moreover, while NOVA measurements are generally confined to major arteries within the Circle of Willis, DNF extends the analytical scope to distal segments. Direct quantitative comparison with NOVA was limited to arterial segments with corresponding measurements, whereas the network-wide distributions, including the relative pressure changes shown in **Figure 8**, were calculated by DNF and are presented as illustrative hemodynamic outputs rather than independently validated predictions or evidence of stenosis-specific diagnostic performance. Provided that small vessels are adequately visualized on MRA-TOF images, the model can reconstruct distal branches and compute their hemodynamic parameters, facilitating a more comprehensive evaluation of the cerebral circulation.

**Figure 8:**
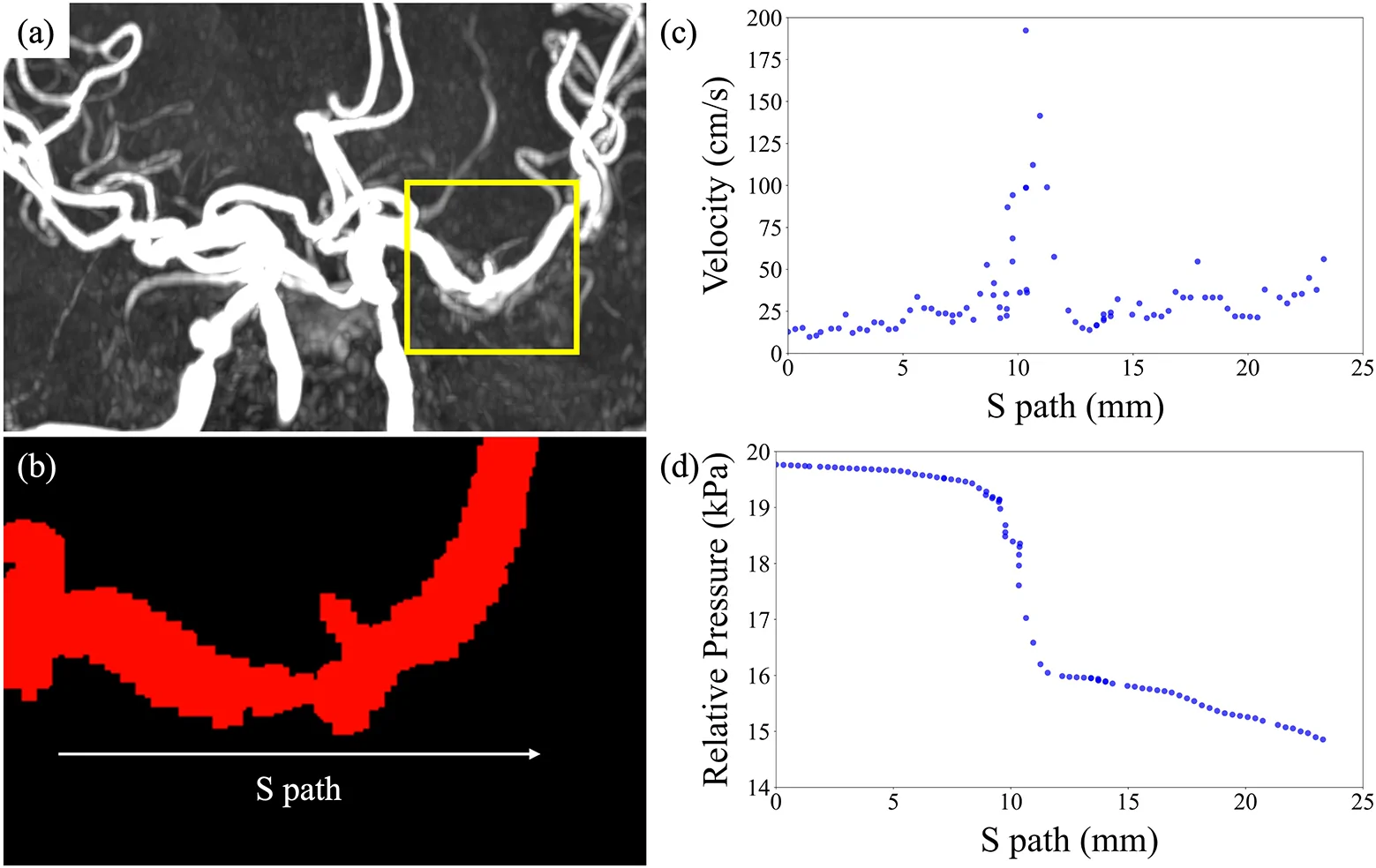
Hemodynamic analysis of a stenotic region in the middle cerebral artery. **(a)** MRA-TOF visualization, **(b)** three-dimensional vessel segmentation, and **(c)** velocity and **(d)** relative pressure changes at stenotic region. Note that the y-axis in **(d)** is truncated to highlight the specific pressure drop across the stenosis.

Subgroup analyses showed that mean flow rates measured by NOVA and predicted by DNF were of similar magnitude across risk-factor groups (**Table 4**). Patient-level random-intercept mixed-effects models showed no significant method-by-risk-factor interactions for diabetes mellitus, dyslipidemia, or hypertension (*p* = 0.548, 0.661, and 0.746, respectively). However, the small size of some reference groups, particularly those without hypertension (n = 8) and without dyslipidemia (n = 12), limited the statistical power to detect modest interaction effects. Accordingly, these subgroup findings should be regarded as exploratory and do not establish comparable performance between NOVA and DNF across all risk-factor groups. Despite this statistical limitation, these cardiometabolic conditions remain physiologically relevant because they can alter cerebral hemodynamics through changes in blood viscosity and vascular structure. For example, diabetes mellitus has been associated with hemorheological abnormalities and structural narrowing of vessels in both the macrocirculation and microcirculation, which may reduce blood flow and increase susceptibility to viscosity-related flow alterations (MacRury and Lowe, 1990). Similarly, hypertension has been reported to increase blood viscosity by up to approximately 28% compared with normotensive conditions across varying shear rates (Letcher et al., 1981). In dyslipidemia, lipid abnormalities have also been linked to changes in blood rheology; high-density lipoprotein has been reported to be inversely associated with blood viscosity, whereas low-density lipoprotein shows the opposite relationship (Sloop and Garber, 1997). However, the relationship between dyslipidemia and blood viscosity is complex and likely mediated by multiple interacting biochemical and metabolic factors (Gyawali et al., 2012). As a conclusion, these conditions are the major components of metabolic syndrome that can complicatedly affect blood vessels and microcirculation on which the blood viscosity depends (Gyawali et al., 2012). Because the Poiseuille equation describes the relationship between pressure gradient, viscosity, vessel radius, and flow rate, such physiological alterations could potentially affect the accuracy of computational flow estimation models such as DNF that rely on these parameters (Çinar, 2001). Although no significant interactions between measurement method and risk-factor status were observed, larger studies incorporating detailed hemorheological and vascular parameters will be necessary to clarify how cardiometabolic risk factors affect patient-specific cerebrovascular flow modeling.

**Table 4:** Subgroup comparison of flow rates measured by NOVA and DNF according to vascular risk factors.

Risk factor	Group	Patients, n	Arteries, n	NOVA (mm³/s)	DNF (mm³/s)	Method × risk *β* [95% CI] (mm³/s)	*p*
Diabetes mellitus	No	16	106	2017.45 ± 1058.16	2025.62 ± 1318.45	−122.80 [−523.24, 277.63]	0.548
	Yes	19	124	1906.32 ± 1087.52	1791.69 ± 1094.00		
Dyslipidemia	No	12	79	1992.62 ± 1260.80	1996.32 ± 1438.51	−94.04 [−514.44, 326.36]	0.661
	Yes	23	151	1939.18 ± 964.73	1848.85 ± 1065.61		
Hypertension	No	8	52	2112.82 ± 1049.73	1993.66 ± 1405.09	78.99 [−398.30, 556.27]	0.746
	Yes	27	178	1912.17 ± 1078.62	1871.99 ± 1143.83		

Values are presented as mean ± standard deviation. *β* denotes the method-by-risk-factor interaction (the between-group difference in DNF−NOVA) estimated using a linear mixed-effects model with a patient random intercept. Values are *β* [Wald 95% CI] and two-sided *p*-values. NOVA, Non-invasive Optimal Vessel Analysis; DNF, Dr. NEAR flow.

Ultimately, DNF has potential as a point-of-care tool that bridges the gap between static anatomical imaging and complex physiological assessment. Its rapid processing capability and comprehensive visualization of the arterial network—including small and tortuous vessels—support its potential for future applications in personalized stroke risk evaluation. Future refinements may include incorporation of patient-specific peripheral resistance parameters based on age or comorbidities (Zhu et al., 2011; Xing et al., 2017), as well as the integration of AI-enhanced segmentation techniques to improve small-vessel radius estimation (Hilbert et al., 2020). In conclusion, the linearized computational framework implemented in DNF provides a feasible approach for cerebral hemodynamic assessment. Further independent validation and prospective evaluation would provide additional evidence on its performance in clinical settings.

## Data Availability

The datasets presented in this article are not readily available because of patient privacy and institutional review board regulations. Requests to access the datasets should be directed to KH, coolghh@gmail.com.
